# Supplementary material for: Familial follicular cell thyroid carcinomas in a large number of Dutch German longhaired pointers
Source: Vet Comp Oncol. 2021 Sep 16;20(1):227–34. doi: 10.1111/vco.12769 (PMC9292937; doi:10.1111/vco.12769)
Supplement: Supplementary file 1 — FIGURE S1 The pedigree of all 264 GLPs with affection information. Circles represent females, and squares represent males. Dot line shows identical dogs. Dogs with FCC histologically diagnosed are highlighted in red, the dog with follicular thyroid adenoma is highlighted in green, and suspected affected dogs are in black, whereas unaffected dogs remain white. A question mark represents the dogs with unknown status. The two rows of texts below the circles or squares represent the ID and diagnosis age (in years), respectively. FIGURE S2 Ancestry family tree of GLP52 and GLP905. These two dogs are half‐first cousins with a common grandfather GLP306. GLP52 was a suspected case. Circles represent females, and squares represent males. Dot line shows identical dogs. The two rows of texts below the circles or squares represent the ID and diagnosis age (in years), respectively. FIGURE S3 Pedigree of GLPs that could be traced back to the cross between GLP296 and GLP319. Circles represent females, and squares represent males. Dot line shows identical dogs. The two rows of texts below the circles or squares represent the ID and diagnosis age (in years), respectively. Dogs with FCC histologically diagnosed are highlighted in red, the dog with follicular thyroid adenoma is highlighted in green, and suspected affected dogs are in black, whereas unaffected dogs remain white. A question mark represents the dogs with unknown status. FIGURE S4 F of affected and unaffected GLPs including the dogs born after 2007 in our dataset (54 cases and 177 controls) (Wilcoxon test, p‐value = 4.317e‐10). Affected dogs are more inbred than unaffected dogs. FIGURE S5 Kinship matrix between 54 FCC cases, one dog with adenoma, 29 suspected cases, and 180 unaffected dogs. Kinsvhip matrix was estimated using kinship2 package in R. The 54 FCC cases are closely related to each other. Meanwhile, most suspected cases are closely related to the histologically diagnosed FCC cases. [file VCO-20-227-s002.docx]

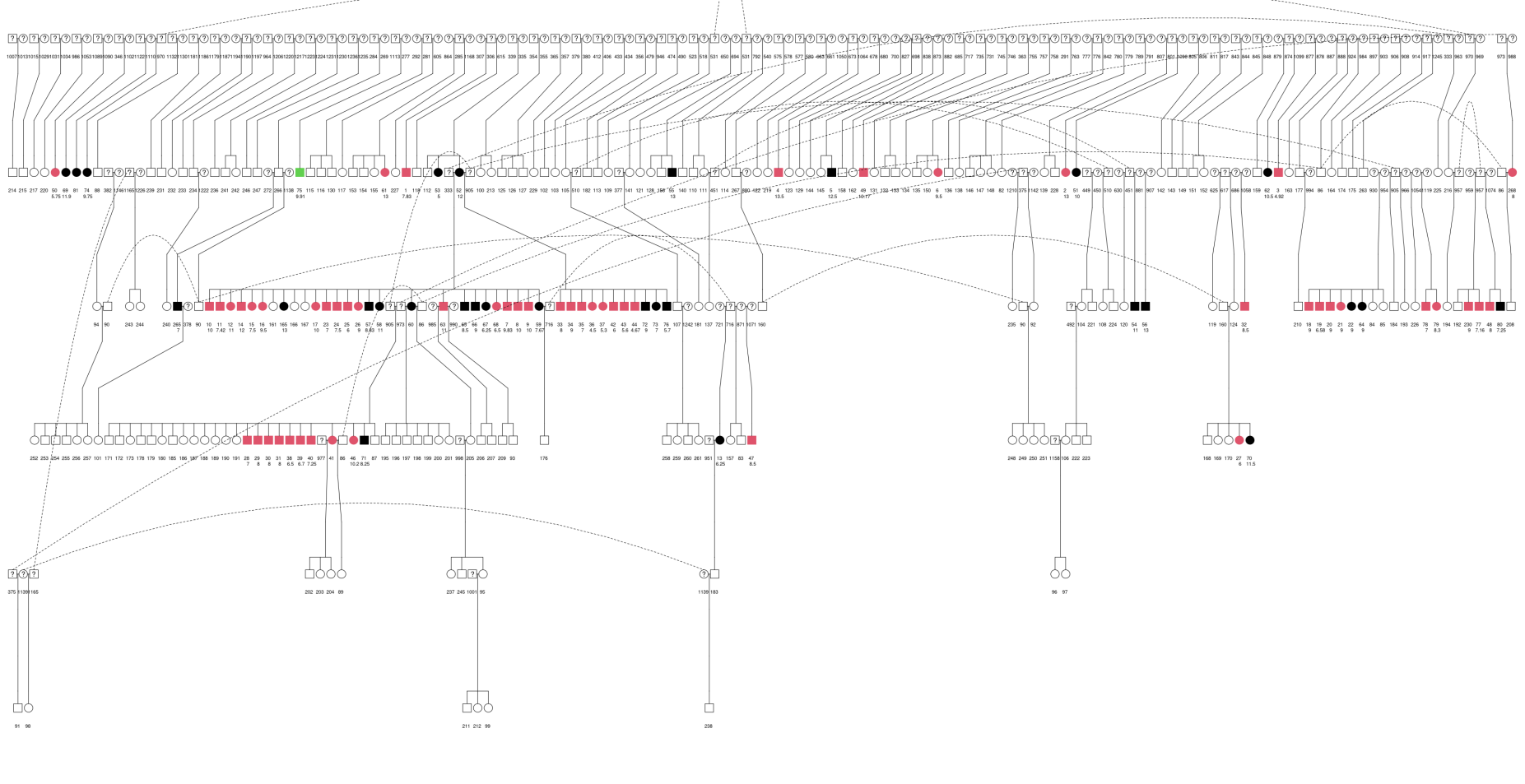


Figure S1. The pedigree of all 264 GLPs with affection information. Circles represent females, squares represent males. Dot line show identical dogs. Dogs with FCC histologically diagnosed are highlighted in red, the dog with follicular thyroid adenoma is highlighted in green, and suspected affected dogs are in black, whereas unaffected dogs remain white. A question mark represents the dogs with unknown status. The 2 rows of texts below the circles or squares represent the ID and diagnosis age (in years), respectively.


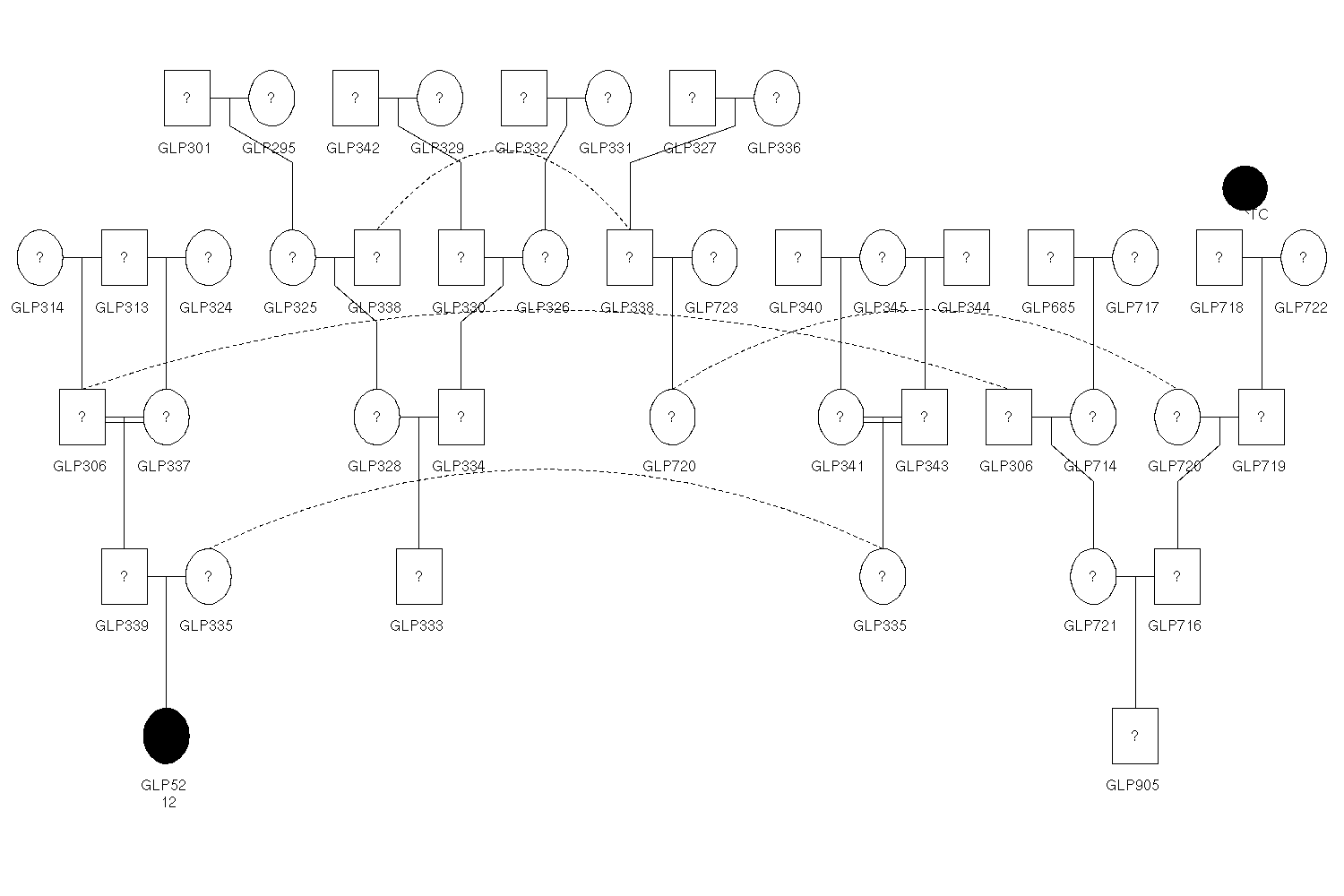


Figure S2. Ancestry family tree of GLP52 and GLP905. These two dogs are half-first cousins with a common grandfather GLP306. GLP52 was a suspected case. Circles represent females, squares represent males. Dot line show identical dogs. The 2 rows of texts below the circles or squares represent the ID and diagnosis age (in years), respectively.


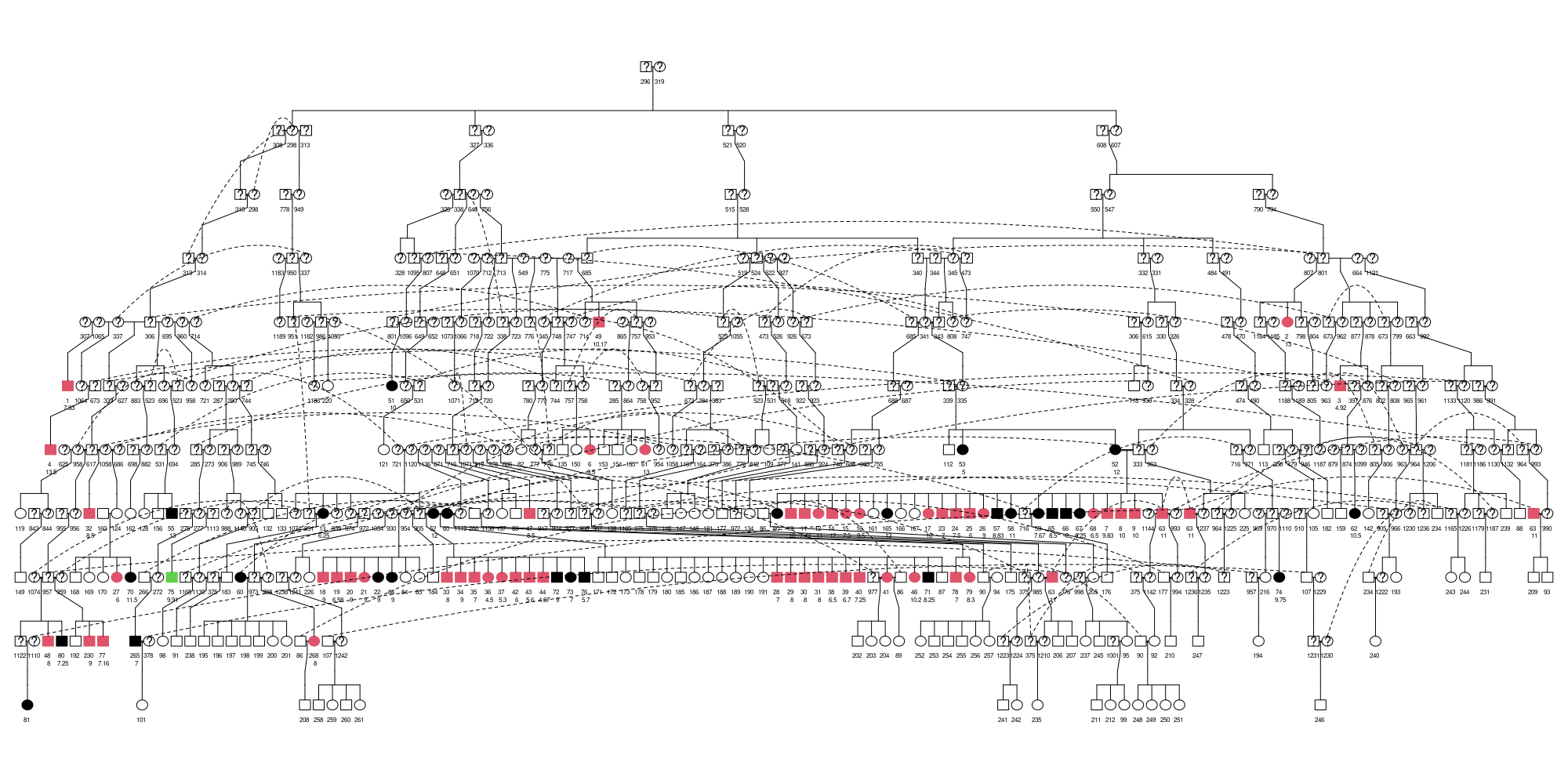


Figure S3. Pedigree of GLPs that could be traced back to the cross between GLP296 and GLP319. Circles represent females, squares represent males. Dot line show identical dogs. The 2 rows of texts below the circles or squares represent the ID and diagnosis age (in years), respectively. Dogs with FCC histologically diagnosed are highlighted in red, the dog with follicular thyroid adenoma is highlighted in green, and suspected affected dogs are in black, whereas unaffected dogs remain white. A question mark represents the dogs with unknown status.


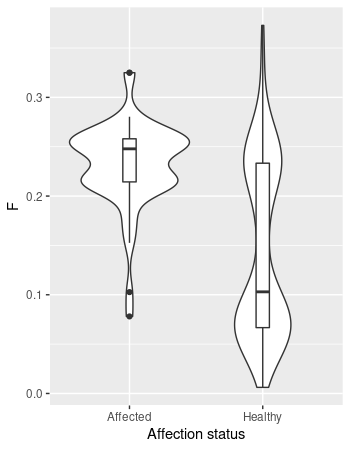


Figure S4. *F* of affected and unaffected GLPs including the dogs born after 2007 in our dataset (54 cases and 177 controls)(Wilcoxon test, *p*-value=4.317e-10). Affected dogs are more inbred than unaffected dogs.


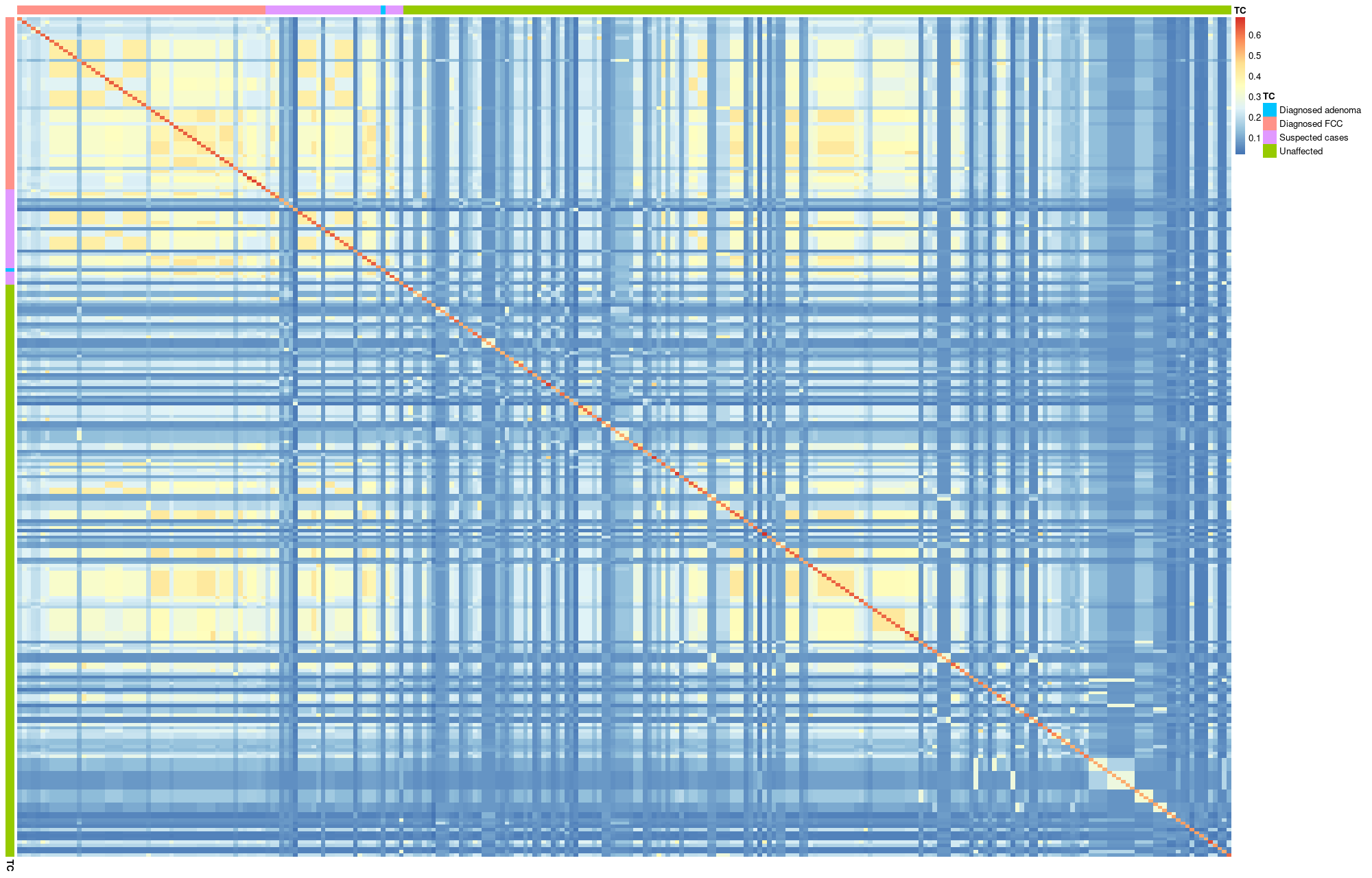


Figure S5. Kinship matrix between 54 FCC cases, 1 dog with adenoma, 29 suspected cases, and 180 unaffected dogs. Kinsvhip matrix was estimated using kinship2 package in R. The 54 FCC cases are closed related to each other. Meanwhile, most suspected cases are closely related to the histologically diagnosed FCC cases.
